# Racial, Ethnic, and Immigration-Related Disparities Across the Gastrointestinal Care Continuum: A Scoping Review

**DOI:** 10.3390/jcm15186961

**Published:** 2026-09-08

**Authors:** Rhea Thomas, Mariam Belghiti, Hiu-Ki Rachel Tran, Parul Tandon

**Affiliations:** Division of Gastroenterology and Hepatology, University Health Network, University of Toronto, Toronto, ON M5S 1A1, Canada; rhea.thomas@medportal.ca (R.T.); mariam.belghiti@mail.utoronto.ca (M.B.); rachel.tran@medportal.ca (H.-K.R.T.)

**Keywords:** gastrointestinal diseases, healthcare access, health disparities, racial minorities, ethnic minorities, immigrant, treatment delay, time to treatment, referral delay

## Abstract

**Background:** Gastrointestinal (GI) diseases contribute substantially to healthcare utilization and chronic disease burden through recurrent symptoms, ongoing specialist care, and diagnostic evaluation. However, disparities related to race, ethnicity, and immigration status may arise at multiple points along the GI care continuum, and their association with access, diagnosis, treatment, and outcomes remains incompletely characterized. This scoping review synthesizes evidence on these differences in GI healthcare delivery. **Methods:** MEDLINE and Embase were searched from 1 January 2000 to 17 June 2025, with supplementary grey literature searches of relevant GI, liver, surgical, and endoscopic society websites. Findings were synthesized thematically due to heterogeneity in study design, populations, outcomes, and reporting. **Results:** Seventy-four studies were included across various GI diseases, including inflammatory bowel disease, hepatobiliary disease, esophageal disorders, and liver transplantation. Differences in care were reported across multiple care stages, including specialist access; diagnostic and treatment delays; use of medical, procedural, and surgical therapies; emergency care reliance; and transplant access and outcomes. Findings were, however, heterogeneous, with some studies reporting no differences or higher utilization among minority groups. **Conclusions:** Racial, ethnic, and immigration-related differences are reported across GI care pathways. Future research should identify modifiable factors that may lead to practice-changing interventions that will improve timely, equitable, longitudinal care.

## 1. Introduction

Gastrointestinal (GI) diseases encompass a broad spectrum of disorders affecting the digestive tract, as well as other accessory organs such as the liver and biliary system. These conditions include disorders of the gut–brain interaction, functional GI disorders such as irritable bowel syndrome (IBS), as well as structural or inflammatory diseases including inflammatory bowel disease (IBD), hepatobiliary diseases, and esophageal disorders [1]. The diagnosis and management of GI diseases are often complex and require multidisciplinary care with treatment strategies ranging from medical therapy to endoscopic and surgical interventions [1]. Consequently, GI diseases represent a major source of healthcare utilization and burden.

The healthcare burden associated with GI diseases is substantial and continues to increase. In the United States, GI conditions contribute significantly to healthcare utilization, with approximately 14.5 million emergency department visits and 2.9 million hospital admissions annually [2]. Globally, non-malignant digestive diseases represent a growing chronic disease burden, accounting for a substantial proportion of non-communicable diseases and contributing significantly to disability-adjusted life years (DALYs) [3]. Delayed diagnosis further exacerbates this burden, increasing the risk of malignancy and negatively impacting quality of life [4]. As such, understanding factors that influence access to timely diagnosis and care—particularly across racial, ethnic, and socioeconomically diverse populations—remains critical.

Despite recent advances, disparities in GI healthcare delivery and outcomes remain a concern, driven by the intersection between race, ethnicity, and key social determinants of health [5,6,7]. These non-clinical determinants represent up to 80% of modifiable influences that occur outside of direct clinical care [8]. Across multiple GI conditions, studies have demonstrated that minority populations experience lower rates of therapy utilization and vaccination, longer wait times, and worse overall outcomes [6,9,10,11,12,13,14]. However, the existing literature remains limited and heterogeneous, with some studies identifying significant disparities in care and outcomes, while others report inconsistent or negligible differences [6,15,16,17,18,19]. Accordingly, a clearer synthesis of the impact of race, ethnicity, and social determinants of health on GI disease outcomes is needed to better define the scope and drivers of these disparities.

We aimed to systematically examine racial and ethnic disparities in GI healthcare delivery. Specifically, we aimed to characterize differences in healthcare utilization, access to care, treatment patterns, and clinical outcomes with respect to race, ethnicity, and immigration status.

## 2. Materials and Methods

### 2.1. Search Strategy

This scoping review was conducted in accordance with the Preferred Reporting Items for Systematic Reviews and Meta-Analyses extension for Scoping Reviews (PRISMA-ScR) guidelines [20]. A comprehensive literature search was conducted in MEDLINE (via Ovid) and Embase from 1 January 2000 to 17 June 2025. The search strategy incorporated both controlled vocabulary (MeSH and Emtree terms) and free-text keywords related to racial, ethnic, or immigrant status, combined with terms for GI diseases. The disease types included in the search were as follows: gastrointestinal, liver, hepatobiliary, esophageal and peritoneal disease, as well as abdominal pain. Additional search terms addressed access to care and healthcare disparities, including wait times, time to treatment, and door-to-treatment time. Grey literature searches were conducted within the same time frame using PubMed and ProQuest, as well as relevant governmental, national, and international organizational websites.

### 2.2. Eligibility Criteria and Screening

We included English-language studies if they were primary research studies, included patients over the age of 18, examined GI diseases, focused on minority populations (e.g., racial, ethnic, immigrant populations), and reported at least one a priori-determined outcome related to access to care, such as wait time, time to diagnosis, time to treatment, healthcare utilization, therapy utilization, or mortality. Population characteristics were classified as follows: immigration status referred to nativity or citizenship relative to the study location, whereas race and ethnicity were used to describe participant groups when referring to racial and cultural origin.

Exclusion criteria included case reports, editorials, and review articles; studies with fewer than 10 patients; pediatric-only populations; non-human studies; qualitative studies; and studies focused exclusively on malignancies to reduce heterogeneity, given the distinct diagnostic and treatment pathways associated with cancer care.

**Figure A1:**
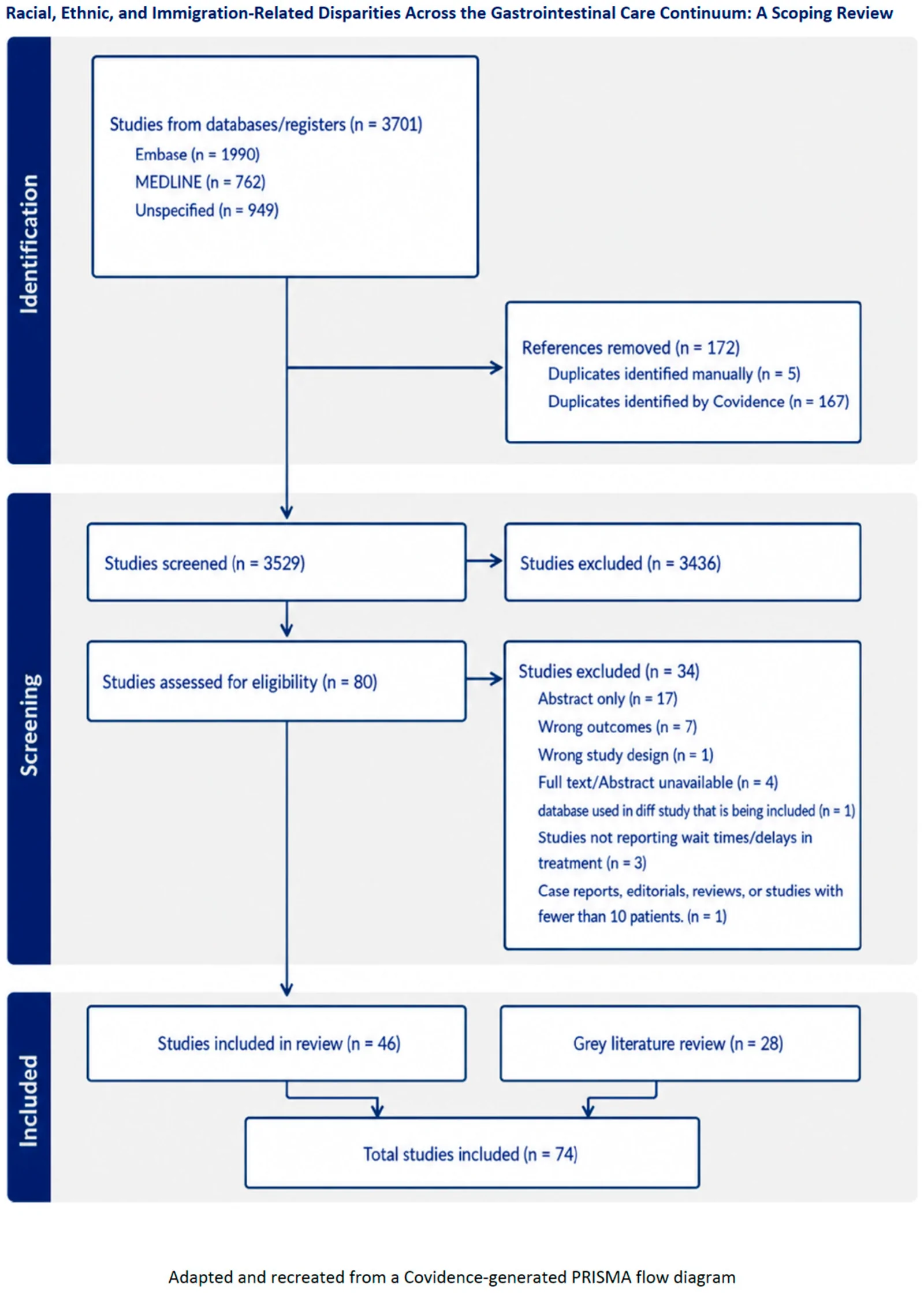
PRISMA-ScR flow diagram of the study screening process and inclusion.

Three independent reviewers (R.T., M.B., H.T.) screened studies in duplicate, starting with title and abstract review followed by full-text review. Discrepancies were resolved by majority consensus, with final adjudication by the principal investigator when required (P.T.). A total of 3529 studies underwent title and abstract screening; 80 progressed to full-text review, and 46 met inclusion criteria and were included for data extraction. The review was supplemented by grey-literature searches of relevant gastroenterology, hepatology, surgical, and endoscopic society websites, resulting in 28 additional papers included for data extraction. A total of 74 papers were included in this scoping review; 61 (82.4%) of which were peer-reviewed full-text journal articles, 12 (16.2%) were conference abstracts, and one (1.4%) was a full-text dissertation identified through the grey-literature search. The study screening process is presented in a PRISMA-ScR flow diagram (Figure A1).

### 2.3. Outcomes and Data Extraction

Data extraction was performed independently by three reviewers (R.T., M.B., H.T.). Extracted variables included study characteristics (year, study continent, and sample size), population demographics (race, ethnicity, immigrant status), GI disease type, reported outcomes, and statistical measures (including odds ratios (OR), hazard ratios (HR), confidence intervals (CI), and *p*-values).

Data extraction occurred in two phases. In the first phase, studies were categorized descriptively by disease type and demographic characteristics, including specifications of the populations studied. In the second phase, extracted outcomes were analyzed according to emergent themes, including healthcare utilization, time to treatment, time to diagnosis, therapy utilization, mortality, and social determinants of health. Studies were organized by both disease category and thematic grouping.

### 2.4. Data Analysis/Themes

Given the heterogeneity in definitions of access-related outcomes, measurement approaches, statistical reporting, and population sizes, formal quantitative synthesis and meta-analysis were not performed. This was determined a priori. Accordingly, findings were synthesized using a scoping review framework. Studies were grouped based on both disease type and outcome measured. Broad thematic categories were developed to accommodate variation in reporting and included therapy utilization, treatment delay, time to treatment, outpatient services, healthcare utilization, socioeconomic and social determinants of health factors, diagnostic utilization, and mortality. Themes were intentionally broad to capture patterns of disparities across diverse GI conditions and their associated outcomes in racial, ethnic, and immigrant populations.

## 3. Results

A total of 74 studies were included in this study (Table 1). The largest number of studies examined inflammatory bowel disease (*n* = 24) and liver transplantation (*n* = 23), followed by hepatitis (*n* = 16), biliary disease (*n* = 4), esophageal disorders (*n* = 2), disorders of the gut–brain interaction (DGBI) (*n* = 4), and acute GI disease (*n* = 2). One study examined two disease categories; therefore, the disease-specific counts are not mutually exclusive. Most studies were conducted in the United States and used retrospective cohort designs, although prospective cohort, cross-sectional, and mixed-methods studies were also included. The populations most commonly compared included White, Black, Hispanic, Asian, Indigenous, and immigrant populations.

### 3.1. Inflammatory Bowel Diseases (IBD)

#### 3.1.1. Therapy Utilization

Fifteen studies evaluated racial and ethnic differences in the utilization of IBD therapies, including medical, surgical, and supportive interventions. When examining therapy utilization, Black patients were less likely than White patients to receive conventional therapies (e.g., thiopurines, amino salicylates, methotrexate), less likely to receive advanced biologics, less likely to receive prolonged steroid therapy, and less likely to receive early disease-modifying therapy within 6 months of diagnosis, with differences persisting up to 10 years [22,24,28,60,64,67,77,85]. Similarly, Hispanic, Asian and South Asian patients demonstrated lower claims for both conventional and advanced therapies, including reduced steroids, immunomodulators, and biologic utilization [28,40,77]. In contrast, some studies reported higher utilization among racial and ethnic groups, with Black patients more likely to receive immunomodulators, anti-TNF therapy, combination therapy, and prednisone, and Hispanic patients demonstrating similar or higher biologic use compared with White patients [24,25,44,49]. Only two studies found no significant differences in therapy use across racial/ethnic groups [54,93].

When examining procedural and surgical interventions, Black, Hispanic, and Asian patients were less likely to undergo colectomy, bowel resection, or abdominal surgery compared to White patients [22,60,66]. Other studies demonstrated that Black and Asian patients were more likely to undergo endoscopic procedures and non-elective procedures/surgeries, and Hispanic patients were more likely to undergo specific surgical procedures such as Abdominoperineal Resection (APR), Lower Anterior Resection (LAR), and Hartmann operations [60,64,85]. One study found no consistent differences in endoscopic utilization [93].

#### 3.1.2. Time to Diagnosis or Treatment

Eight studies evaluated racial and ethnic differences in time to diagnosis and time to treatment across multiple stages of care. When examining time to treatment and authorization, Black patients had longer median time to initiation of both conventional and advanced therapies, and longer time to authorization compared with White patients, with some evidence also suggesting longer time to diagnosis [21,28,29,71]. Hispanic and Asian patients similarly demonstrated delays in treatment initiation, particularly for advanced therapies compared to White patients [28]. Across groups, delays to treatment were most pronounced among Black patients compared with Hispanic and Asian patients [21].

In contrast, some studies reported earlier or equivalent treatment timelines, with Black patients more likely to initiate anti-TNF monotherapy earlier than White patients, though no differences were observed for immunomodulator or combination therapies [25]. Other studies found no significant racial or ethnic differences in time to first biologic infusion, time to diagnosis, or time from diagnosis to surgery [33,63,71].

When examining immigrant status, overall time to diagnosis did not differ significantly between immigrants and non-immigrants with IBD [26]. However, subgroup analyses demonstrated longer time to diagnosis among patients with ulcerative colitis from Latin America or the Caribbean compared to non-immigrant individuals [26].

#### 3.1.3. Healthcare Utilization

Twelve studies evaluated racial and ethnic differences in healthcare utilization among patients with IBD. When examining healthcare utilization and hospitalization outcomes, Black patients had significantly more IBD-related ED visits, higher emergency medical service use, greater hospitalization rates, increased inpatient service use prior to diagnosis, higher readmission rates, more non-elective procedures, greater odds of an extended hospital length of stay, and increased all-cause mortality, with differences observed both early and up to 10 years following diagnosis compared with White patients [22,42,49,60,77,85]. Immigrants and Asian patients similarly demonstrated increased ED visits and persistently higher hospitalization rates at long-term follow-up [26,60]. Hispanic patients were also more likely to undergo emergency surgery and had higher odds of readmission compared with White patients [64].

Other studies demonstrated no significant differences in hospitalization rates or ED utilization between racial and ethnic groups [67,69], while others demonstrated lower ED visits and hospital admissions among Hispanic patients compared with White patients or no significant differences in clinic visits, ED visits, or hospitalizations despite a longer length of stay [44,49]. Similarly, some studies reported no differences in length of stay between Black, Hispanic, and Asian patients compared to White patients [85,93].

When assessing patient-reported outcomes and quality of life, patients from racial and ethnic groups reported more uncontrolled symptoms and lower quality of life across multiple domains [78]. However, in the same study, access to medications, clinicians, and understandable explanations did not differ across racial and ethnic groups.

#### 3.1.4. Outpatient Services and Linkage to Care

Five studies evaluated racial and ethnic differences in outpatient services and linkage to IBD care. When examining linkage to gastroenterology care and specialist access, Black patients were less likely to see a gastroenterologist at least once per year, less likely to be followed by an IBD subspecialist, less likely to report an IBD specialist as their primary provider, and more likely to report difficulty obtaining specialist referrals and concerns regarding healthcare-related costs [67]. Asian patients were similarly less likely to have seen a gastroenterologist, including among older adults [77]. Patients from racial and ethnic groups in general also reported greater difficulty accessing IBD specialists, more frequent reliance on emergency department services, and poorer access to emotional and community support [78].

One study reported no differences in outpatient visit frequency, with Black and White patients having similar numbers of IBD-related ambulatory visits over a 12-month period [67], while another demonstrated longer follow-up duration among Black patients compared with White patients [25]. In contrast to reduced access observed in other groups, Canadian immigrant populations demonstrated higher outpatient healthcare utilization, including more IBD-specific and IBD-related visits, and were more likely to see a gastroenterologist, with greater specialist involvement associated with a reduced likelihood of surgery [26].

#### 3.1.5. Mortality/Morbidity

Only one study reported death and morbidity in IBD patients, with results showing that Black patients were at significantly increased risk of death and serious morbidity compared to White patients [64].

Table 2 summarizes the results of studies exploring racial and ethnic differences in the management of inflammatory bowel disease.

### 3.2. Hepatobiliary Disease

#### 3.2.1. Time to Treatment

Three studies evaluated racial and ethnic differences in time to treatment for biliary disease. Black patients were more likely to experience longer total adjusted wait times and longer times to surgery for both elective and non-elective cholecystectomies, while Hispanic patients similarly experienced longer wait times, including higher odds of delays beyond 90 days and longer overall time to surgery [70,79,80]. In contrast, White patients consistently had the shortest wait times across both elective and non-elective procedures for symptomatic cholelithiasis [79,80].

#### 3.2.2. Healthcare Utilization

Four studies evaluated racial and ethnic differences in healthcare utilization among patients with biliary disease, including emergency department (ED) utilization, readmissions, hospital length of stay, and perioperative outcomes. When examining ED utilization and hospitalization outcomes, Black patients were more likely to undergo unplanned reintubation, remain hospitalized beyond 30 days, have longer overall and postoperative lengths of stay, experience longer intervals between discharge and readmission, have lower rates of intraoperative cholangiography, be more likely to undergo emergent procedures, have longer operative times, and have higher odds of requiring additional ED visits for both non-elective and elective cholecystectomy [70,79,80]. Black and Hispanic patients were also more likely to have repeat ED visits prior to non-elective cholecystectomy compared with White patients [79,80].

Other studies found no significant differences in reoperation or unplanned readmission rates between Black and White patients with acute cholecystitis [70], while others demonstrated that White patients accounted for the majority of hospitalizations for primary biliary cholangitis (PBC), with all racial and ethnic groups experiencing increasing hospitalization rates for PBC over time [43].

#### 3.2.3. Mortality/Morbidity

With respect to biliary-disease-specific mortality, there were inconsistent effects observed, with one study demonstrating no difference in morbidity or mortality between Black and White patients [70], whereas another demonstrated significantly higher in-hospital mortality in Black patients with PBC compared to White individuals [43].

Table A1 summarizes the results of studies exploring racial and ethnic differences in the management of hepatobiliary disease.

### 3.3. Disorders of the Gut–Brain Interaction (DGBI)

Four studies evaluated racial and ethnic differences in care delivery and outcomes among patients with disorders of the gut–brain interaction (Table 3). Hispanic, Black, and Asian patients with IBS were significantly less likely to receive gastroenterology consultation despite higher rates of primary care utilization and were less likely to report that their symptoms were adequately addressed [56,81]. Non-White patients also reported more severe abdominal pain yet were less likely to receive analgesia or opioids, experienced longer wait times to treatment, and demonstrated a higher likelihood of delayed clinical assessment compared with White patients [61,82]. Finally, Hispanic, Black, and Asian patients were more likely to undergo gastroenterology-related procedures, including colonoscopies and upper endoscopies, compared with White patients [81].

### 3.4. Esophageal Disorders

Two studies evaluated racial and ethnic differences in outcomes among patients with esophageal disorders, including treatment delay and treatment utilization [41,92]. Black, Hispanic, and other ethnic patients were more likely to delay care for self-reported dysphagia, primarily due to longer wait times to see a gastroenterologist rather than cost-related barriers [92]. South Asian patients with achalasia were also more likely to undergo surgical procedures such as Peroral Endoscopic Myotomy (POEM) and Heller’s myotomy, and less likely to receive Botulinum toxin therapy compared with White patients, with no observed differences in pneumatic dilatation [40].

Table A2 summarizes the results of studies exploring racial and ethnic differences in the management of esophageal disorders.

### 3.5. Acute Gastrointestinal Diseases

Two studies examined the role of race in the time to diagnosis for acute appendicitis and general GI disease. White patients were more likely to present with private insurance compared to non-White patients, and this led to significant delays in diagnosis and longer lengths of hospital stays for the latter group presenting with acute appendicitis [27]. Similarly, Hispanic patients, compared with non-Hispanic individuals, had longer wait times in the ED for acute GI diseases characterized by conditions such as acute pancreatitis, cholecystitis, diverticulitis, and upper GI hemorrhage [82].

Table A3 summarizes the results of studies exploring racial and ethnic differences in the management of acute gastrointestinal diseases.

### 3.6. Hepatitis

#### 3.6.1. Therapy Utilization

Nine studies evaluated racial, ethnic, and immigration-related differences in hepatitis therapy utilization. Immigrant, Black, Hispanic, and Asian patients were less likely to receive antiviral therapy, including lower overall treatment rates, lower treatment initiation, lower rates of treatment-related monitoring and longer delays from linkage to care to treatment initiation [39,46,50,62,90]. These differences persisted across treatment eras and despite overall increases in treatment uptake over time, with lower odds of treatment observed among Hispanic and Black patients in both pre- and post-DAA periods and longer time to treatment initiation among African American patients compared with White patients [46,50]. Additionally, geographic variation was observed, with Asian patients in Western regions less likely to receive nucleos(t)ide analogues compared with Asian patients in Eastern regions, while no regional differences were observed among non-Asian patients [52].

Other studies demonstrated no significant differences in treatment initiation or treatment types across racial, ethnic, or immigrant groups [36]. There was also no association found between duration of residence and treatment initiation, and no overall differences in treatment rates by ethnicity [45,52]. Additionally, some groups demonstrated higher treatment uptake, with Asians and Native Americans more likely to receive treatment compared with White patients [13].

#### 3.6.2. Diagnostic Utilization

Eight studies evaluated racial, ethnic, and immigration-related differences in diagnostic utilization among patients with hepatitis. Immigrant, Black and Asian patients were less likely to receive an adequate diagnostic evaluation, including routine laboratory monitoring, DNA quantification, and liver biopsies, compared with non-immigrant or non-racial patients [52,62,88,90].

Other studies demonstrated higher diagnostic utilization among immigrant populations, including greater use of FibroScan compared with Canadian-born patients [36], as well as higher rates of testing among foreign-born Asian/Pacific Islander individuals compared with U.S.-born counterparts [48].

#### 3.6.3. Healthcare Utilization

Two studies evaluated racial and ethnic differences in healthcare utilization among patients with hepatitis, with findings demonstrating variation in utilization patterns across groups. Hispanic patients had higher total hospital costs compared with both White and Black patients, while Black patients demonstrated lower inpatient utilization, including lower costs and fewer procedures; untreated individuals also had higher proportions of emergency encounters compared with treated patients [39,59].

Other studies demonstrated that White patients had longer lengths of stay and higher numbers of inpatient procedures compared with Black patients, while utilization patterns between White and Hispanic patients were similar for certain measures [59].

#### 3.6.4. Linkage to Care

Six studies evaluated racial, ethnic, and immigration-related differences in linkage to care and follow-up among patients with hepatitis. Black patients were less likely to achieve linkage to care compared with non-African or Asian patients. Immigrant patients were more likely to be lost to follow-up within the first year, and untreated individuals were less likely to have specialty care involvement [39,53,86,90].

Other studies demonstrated higher linkage to care among Asian patients compared with non-Asian patients, while others found no significant differences in linkage or retention to care across racial and ethnic groups, no long-term differences in follow-up between immigrant and non-immigrant populations, and earlier presentation to care among foreign-born individuals compared with non-immigrant populations [30,46,86,90].

#### 3.6.5. Mortality

Two studies evaluated racial and ethnic differences in mortality outcomes, particularly among patients with hepatitis. Black patients had the highest probability of waitlist mortality compared with other racial groups across multiple time points [31]. Asian patients had lower waitlist mortality compared with White patients when all-cause deaths were considered [31,59].

Table 4 summarizes the results of studies exploring racial and ethnic differences in the management of hepatitis.

### 3.7. Liver Transplantation

#### 3.7.1. Diagnostic and Therapy Utilization

Two studies identified differences in diagnostic and therapeutic interventions related to liver transplantation. Black patients with cirrhosis were significantly less likely to receive screening for varices, and Black, Asian, and Hispanic patients were all less likely to undergo endoscopic variceal screening within six months of diagnosis compared with White patients [73]. Additionally, Black patients had a significantly lower likelihood of receiving a Trans-jugular Intrahepatic Portosystemic Shunt (TIPS) [84].

#### 3.7.2. Treatment Delay and Time to Therapy

In two studies, Hispanic, Black, and multiracial patients experienced longer delays to referral [34,37], while non-Hispanic ethnicity was significantly more protective of time to diagnosis than Hispanic ethnicity prior to adjustment, after which this was no longer significant [37]. After adjustment, the time to cirrhosis diagnosis was significantly greater for multiracial patients [37].

#### 3.7.3. Healthcare Utilization

Two studies investigated the association between healthcare utilization, in the context of liver transplantation, and race, ethnicity, and immigration status. Black patients experienced shorter stays in hospital but lower rejection rates for liver transplantation compared to White patients [83]. Conversely, Indo-Asian patients had longer hospital stays [74]. Black patients also had significantly fewer platelet transfusions, though no differences existed for other blood products [83].

#### 3.7.4. Outpatient Services

Two studies investigated differences in outpatient services related to liver transplantation, where follow-up was similar between Black and White patients [83]. Hispanic patients had decreased chances of being referred to palliative care [76].

#### 3.7.5. Rate of Transplantation

Twenty-one studies evaluated racial and ethnic differences in liver transplantation, including rates of transplantation, waitlisting, and waitlist outcomes. Hispanic, Black, Native American, and First Nations patients were less likely to receive liver transplantation compared to White patients, including lower transplant-to-listing ratios, lower overall likelihood of transplantation, lower likelihood of living-donor liver transplant, lower waitlisting rates, and higher risk of waitlist removal due to deterioration or death, while White patients had the highest transplant volumes and were overrepresented on waitlists relative to the general population [32,35,38,47,51,57,58,68,72,87,91]. Differences in access were evident early in the care pathway, with patients from racial and ethnic groups underrepresented in waitlisting and more likely to be denied listing for psychosocial reasons, despite some groups comprising a higher proportion of transplant recipients relative to their waitlist representation [38,91]. These differences were further exacerbated during the COVID-19 pandemic, with the same patients experiencing higher waitlist inactivation and reduced transplant rates compared with White patients [55].

Some studies demonstrated no significant differences in transplant rates across racial and ethnic groups [32,51,83,89], while others reported higher transplant-to-listing ratios among Black patients compared with other groups [75,87].

#### 3.7.6. Mortality

Sixteen studies evaluated racial and ethnic differences in mortality associated with liver transplantation, including waitlist mortality, listing-to-death ratios, and post-transplant survival, with findings demonstrating variation across groups. First Nations, Hispanic, and Black patients had higher mortality across the transplant pathway, including higher waitlist mortality, lower listing-to-death ratios, and greater likelihood of death or clinical deterioration prior to transplant, with Black patients also experiencing increased waitlist removal due to death during the COVID-19 pandemic [23,32,35,51,55,72]. Once discharged from hospital following liver transplantation, Black patients were found to have the shortest survival, although this was not true for non-transplanted patients [84].

Other studies demonstrated lower waitlist mortality among Black patients and no differences in waitlist mortality among Hispanic patients compared with White patients [57,65], while others reported higher listing-to-death ratios and improved post-transplant survival among Asian and Hispanic patients compared with White patients [51,75,87], no differences in post-transplant mortality between racial groups, and shorter post-transplant survival among White patients compared to Black patients [74,83].

Table 5 summarizes the results of studies exploring racial and ethnic differences in access to and outcomes of liver transplantation.

## 4. Discussion

The diagnosis and management of chronic GI disorders necessitate early detection, proper specialist consultation, and long-term follow-up. This review suggests that racial, ethnic, and immigration-related differences occur across the GI care continuum, rather than being limited to a single disease or outcome (Table 1). In particular, the strongest evidence of these differences existed in the context of therapeutic service utilization [31,73], delays in accessing treatment [20,46,56,61,66,71,78,79,90], high utilization of acute care [22,48,62,67,69,70,78,79], and transplantation services (Table 5), with minority patients more frequently experiencing lower use of and access to resources in these domains and greater reliance on emergency or inpatient care. Smaller bodies of evidence also suggested delays in specialist evaluation [28,69,85], symptom management [56], and diagnostic assessment [56,61]. Across studies, these findings highlight important inequities in healthcare utilization patterns, health system structures and the broader structural contexts associated with race, ethnicity, and immigration status [94].

The findings of this study suggest that the earliest differences appear to occur prior to entry into specialist care. Although findings were heterogeneous, several studies identified early pathway differences in outpatient access and linkage to care [28,69,74,85], diagnostic utilization [27,31,45,50,90], and time to diagnosis across several GI diseases [20,41,63]. Collectively, the included studies suggest that racial and ethnic populations experience reduced specialist access, greater referral barriers, delayed diagnostic evaluation, and fragmented linkage to longitudinal care. Similar differences were reported among immigrants, defined by nativity and citizenship relative to the study location, which may reflect differences in how individuals are perceived or treated within healthcare systems outside their country of origin [27,63,90,95,96,97,98]. Taken together, such inequities in GI outcomes may be shaped by delays and barriers early in the care pathway, such as lower gastroenterology or subspecialist involvement, incomplete diagnostic workup, longer referral times, and longer ED wait times [34,41,84]. These early barriers may be exacerbated by issues related to patients’ access to interpreter services, difficulties in using referral processes, diagnostic testing delays due to systemic biases, availability of appointments, and providers’ lack of awareness of relevant disease screening guidelines [95,96,97,98].

Additionally, several studies identified treatment-stage differences in time to treatment [20,21,70,71,78,79], therapy utilization [21,22,24,25,40,44,49,54,60,64,66,71,77,85,93], and procedural or surgical care across GI diseases [22,27,28,34,35]. Racial, ethnic, and immigrant populations experienced lower access to therapeutic interventions [27,31,73], likely shaped by limited access to disease-modifying medications, procedural interventions, surgical management, and transplant-related care. These treatment-stage differences may continue to reflect similar barriers in early care pathways, such as insurance restrictions, authorization processes, language and communication barriers, health literacy, and patient trust, in addition to a greater emphasis on inequitable access to subspecialty care [94,99]. For example, a recent study suggested that when minority patients have access to appropriate specialty care and disease-specific therapy, previously observed disparities in treatment, such as medication use and surgery utilization, may be reduced or no longer observed [24]. Assumptions about treatment acceptability and limited culturally adapted services may also contribute to disparities in treatment stages [100].

When early access and treatment barriers are not addressed, differences then become visible through patterns of healthcare utilization, which may serve as a downstream marker of delayed or fragmented GI care. While also heterogeneous, several studies revealed that racial, ethnic, and immigrant populations experienced greater reliance on acute, emergency, non-elective, and inpatient services instead of planned outpatient management [24,26,40,63,64,67,70,78,79,85]. Increased healthcare utilization may reflect a shift from preventive, longitudinal, and elective care toward crisis-driven care, driven by upstream barriers related to disease burden, inadequate symptom control, and social determinants of health [101]. Other mechanisms underlying these utilization patterns were not consistently evaluated in the included studies. However, broader literature suggests that there may also be components of stigma, cultural differences, discomfort addressing psychosocial contributors to symptoms, communication barriers, and clinician uncertainty, which can contribute to delayed diagnosis, incorrect management, repeated referrals, or greater reliance on downstream care [102,103,104]. Components of systemic, overt, and unconscious racism likely also contribute to disparities in the quality of and access to healthcare [105]. The most severe consequences of these accumulated barriers are seen in advanced-outcome differences, including higher waitlist mortality and shorter post-transplant survival (Table 5). These findings may reflect both more advanced disease at listing or transplant and social or structural barriers to timely transplant access [106,107].

The presented pathway-based interpretation has important clinical and policy implications. The findings of this review suggest that new interventions should target specific points where differences repeatedly appeared. Proposed strategies for clinical and policy improvements should align with broader access recommendations emphasizing telemedicine, community outreach, patient navigation, workforce and procedural capacity investment, and transparent auditing of access and outcomes to reduce differences in GI care [108]. Culturally safe and language-concordant care training may also improve clinician attitudes and knowledge, as well as patient satisfaction and treatment adherence [109,110]. Future research should also move beyond describing disparities to identifying why they occur at specific transition points in the care pathway. Prospective, multicenter studies and disease registries may be particularly useful for clarifying these mechanisms, but should also further investigate how to isolate the implications of race, ethnicity, and immigration status from other social determinants of health and their respective contributions to health outcomes [6]. Immigrant status also requires more careful study, as immigrant populations differ by country of origin, reason for migration, duration of residence, and documentation status.

This study has several strengths and limitations. It examines differences across a broad range of GI conditions and evaluates multiple clinically meaningful stages of care, allowing patterns to be identified beyond a single disease area or stage of the care pathway. Another strength is the inclusion of race, ethnicity, and immigration-related variables, which allowed this review to capture differences affecting multiple historically marginalized populations. The systematic search strategy, duplicate screening process, and thematic synthesis strengthen the comprehensiveness and reproducibility of the review. However, conference abstracts comprised 16.2% of included studies. Compared with peer-reviewed, full-text publications, the abstracts offered limited information on study design and analytic methods. This limited our ability to assess methodological quality, adjustment for confounders, and evaluate the robustness of the reported findings. In addition, reported results may have been subsequently modified, expanded, or not published as full articles, potentially contributing to selective publication and outcome-reporting bias. Furthermore, our interpretation was limited by heterogeneity in study design, healthcare setting, and outcome definitions. Furthermore, many studies were observational or retrospective, restricting causal interpretation and leaving the results vulnerable to confounding factors such as socioeconomic status and disease severity.

Overall, this study demonstrates that marginalized populations experience differences in healthcare access along the GI care pathways. Inequities in access and outcomes are not isolated to a particular disease but reflect broader structural barriers to care that shape how patients enter, move through, and benefit from GI-related healthcare. Future efforts should focus on identifying modifiable barriers, improving culturally safe care, and developing interventions to support timely, equitable, and longitudinal care for marginalized populations.

## Figures and Tables

**Table 1:** Summary of study characteristics included in this scoping review.

Author, Year	Gastrointestinal Disease	Country	Study Type	Patients Included (*n*)	Race/Ethnicities Compared
Abadir 2024 [21]	Inflammatory bowel disease	Canada	Prospective cohort study	97	White, Asian, Black, Latin, Pacific Islander
Alchirazi 2022 [22]	Inflammatory bowel disease	Global	Retrospective cohort study	632,315	White, Black
Ayuk-Arrey 2025 [23]	Liver transplant	United States	Retrospective cohort study	449,211	White, Black, Hispanic, Other
Barnes 2018 [24]	Inflammatory bowel disease	United States	Prospective cohort study	5906	Black, Asian, White
Barnes 2021 [25]	Inflammatory bowel disease	United States	Retrospective cohort study	14,735	Black, White
Benchimol 2016 [26]	Inflammatory bowel disease	Canada	Population-based cohort study	24,192	Immigrant and non-immigrant Canadians
Bickell 2008 [27]	Appendicitis	United States	Retrospective observational study	236	White, Non-White
Branchcomb 2024 [28]	Inflammatory bowel disease	United States & European Union	Cross-sectional chart review/retrospective observational study	27,691	White, Hispanic, Black, Asian, Other
Buadu 2024 [29]	Inflammatory bowel disease	United States & European Union	Cross-sectional study	1833	Hispanic, Black, White, Other
Buti 2020 [30]	Hepatitis (HBV)	Spain	Retrospective registry review	398	Non-Spanish-born and African
Bzowej 2009 [31]	Hepatitis (HBV)/Liver transplant	United States	Retrospective cohort study with prospective follow-up	274	White, Asian, Black
Chahal 2021 [32]	Liver transplant	Canada	Retrospective cohort study	4916	South Asian, Asian, Indigenous, White
Chandradevan 2024 [33]	Inflammatory bowel disease	United States	Retrospective review	98	White, Black, Asian
Chatterjee 2023 [34]	Liver transplant	United States	Retrospective cohort study	363	White, Hispanic, Black
Cholankeril 2018 [35]	Liver transplant	United States	Retrospective cohort study	5472	White, Hispanic, Black, Other
Cooper 2017 [36]	Hepatitis (HCV)	Canada	Retrospective cohort analysis	2335	Black, White, Asian, Aboriginal, Not stated
Cuomo 2024 [37]	Liver transplant	United States	Retrospective cohort study	2974	White, Non-White
Deutsch-Link 2023 [38]	Liver transplant	United States	Retrospective cohort study	2271	Asian, Black, Hispanic/Latinx, Other, White
Elnaiem 2025 [39]	Hepatitis (HCV)	United States	Retrospective cohort study	4345	Black, White
Farrukh 2015 [40]	Inflammatory bowel disease	United Kingdom	Retrospective study	139	European, South Asian
Farrukh 2021 [41]	Esophageal disease	United Kingdom	Retrospective ecological study	1198	White British, South Asian
Frieder 2023 [42]	Inflammatory bowel disease	United States	Retrospective cohort study	38,143	White, Black, Other
Galoosian 2020 [43]	Biliary disease	United States	Retrospective cohort study	18,279	White, Black, Hispanic, Asian
Garcia 2024 [44]	Inflammatory bowel disease	United States	Retrospective observational study	9032	Hispanic and Non-Hispanic
Giordano 2009 [45]	Hepatitis (HCV)	Canada	Retrospective cohort study	910	White, Black, Asian, Aboriginal
Gomes 2020 [46]	Hepatitis (HCV)	United States	Retrospective cohort study	600	White, African American, Hispanic, Asian, Other
Goyes 2021 [47]	Liver transplant	United States	Retrospective cohort study	28,278	White, Hispanic
Hu 2013 [48]	Hepatitis (HBV)	United States	Cross-sectional observational survey study	53,896	Black, Hispanic, Asian/Pacific Islander, American Indian/Alaska Native
Hussain 2025 [49]	Inflammatory bowel disease	United States	Retrospective database cohort study	10,578	non-Hispanic White, Hispanic, non-Hispanic Black, Other
Jiang 2023 [50]	Hepatitis (HCV)	United States	Retrospective cohort study	52,846	White, Black, Hispanic, Native American
Kaplan 2023 [51]	Liver transplant	United States	Retrospective database cohort study	225,504 (CDC WONDER and UNOS databases)	Asian, Black, Hispanic, White
Kudaravalli 2024 [52]	Hepatitis (HBV)	Global	Cross-sectional study	12,566	Asians in East, Asians in West, Non-Asians in East, Non-Asians in West [East (Hong Kong, Japan, Korea, Singapore, and Taiwan) and West (Argentina, Romania, Spain, and the USA)]
Le 2022 [53]	Hepatitis (HBV and HCV)	United States	Retrospective cohort study	8739	African immigrants, Asian immigrants, Black, Hispanic, White, Unknown
Lin 2013 [54]	Inflammatory bowel disease	United States	Cross-sectional study	26,400,000	White, Black, Hispanic, Asian
MacConmara 2021 [55]	Liver transplant	United States	Retrospective cohort study	21,702	White, Minority
Martinez 2023 [56]	Irritable bowel syndrome	United States	Cross-sectional study	518	White, Hispanic
Mathur 2010 [57]	Liver transplant	United States	Retrospective cohort study	39,114	White, Black, Hispanic, Asian, Other
Mathur 2014 [58]	Liver transplant	United States	Retrospective population-level observational study	423,534 (deaths) and 72,245 (wait-list registrations)	Black, Asian, Hispanic, White, Native American/Other
May 2016 [59]	Hepatitis (Alcoholic)	United States	Retrospective cross-sectional study	11,304	White, Black, Hispanic
McFalls 2025 [60]	Inflammatory bowel disease	United States	Population-based retrospective analyses	284,550	White, Black, Asian, Other, Native Hawaiian/Pacific Islander and American Indian or Alaska Native
Mills 2011 [61]	Abdominal pain	United States	Retrospective cohort study	20,125	White, Non-White
Miquel 2020 [62]	Hepatitis (HBV)	Spain	Cross-sectional study	951	Non-immigrant vs. Immigrant
Misra 2019 [63]	Inflammatory bowel disease	United Kingdom	Prospective population-based epidemiological study	339	White European, Indian, Pakistani, Other
Montgomery 2018 [64]	Inflammatory bowel disease	United States	Retrospective cohort study	14,679	White, Black, Asian, Hispanic, Other
Mustian 2019 [65]	Liver transplant	United States	Retrospective cohort study	101,805	White, Black
Nguyen 2007 [66]	Inflammatory bowel disease	United States	Retrospective cohort study	1126	White, Black, Hispanic
Nguyen 2010 [67]	Inflammatory bowel disease	United States	Cross-sectional study	286	Black and White
Nobel 2015 [68]	Liver transplant	United States	Retrospective cohort study	35,401	White, Black, Hispanic, Asian, Other
Odufalu 2024 [69]	Inflammatory bowel disease	United States	Mixed methods, observational study design	1449	White, Asian, Hispanic, Black/African American, American Indian or Alaskan Native, Native Hawaiian or Pacific Islander, Other
Porras Fimbres 2023 [70]	Biliary disease	United States	Retrospective cohort study	121,466	Black, White
Rao 2024 [71]	Inflammatory bowel disease	United States	Retrospective observational study	66	Black, Asian, Other
Reid 2004 [72]	Liver transplant	United States	Retrospective cohort study	29,013	Black, White
Robinson 2018 [73]	Liver transplant	United States	Retrospective cohort study	157	Hispanic, Black, non-Hispanic White, Asian, Other
Rocha 2015 [74]	Liver transplant	United Kingdom	Retrospective matched cohort study	182	Indo-Asian, White
Rosenblatt 2021 [75]	Liver transplant	United States	Retrospective cohort study	216,672	White, Hispanic, Black, Asian
Rush 2017 [76]	Liver transplant	United States	Retrospective nationwide cohort analysis	39,349	White, Black, Hispanic
Segura 2025 [77]	Inflammatory bowel disease	United States	Retrospective cohort study	47,301	Asian, Black, Hispanic, White
Shah 2024 [78]	Inflammatory bowel disease	United States	Cross-sectional survey study	1217	Black/Indigenous/People of Color/Hispanic (BIPOC/H) or White non-Hispanic
Shenoy 2021 [79]	Biliary disease	United States	Retrospective cohort study	35,328	Black, Hispanic, White
Shenoy 2022 [80]	Biliary disease	United States	Retrospective cohort study	13,596	White, Hispanic, Black, Asian, Other
Silvernale 2021 [81]	Irritable bowel syndrome	United States	Retrospective matched cohort study	3823	Hispanic, Black, Asian, White
Simons-Linares 2018 [82]	General gastrointestinal disease	United States	Cross-sectional study	16,999,442	White, Black, Hispanic
Smith 2017 [83]	Liver transplant	United States	Retrospective cohort study	913	Black, White
Solano 2024 [84]	Liver transplant	United States	National cohort study	5915	Black, Hispanic, White
Syal 2017 [85]	Inflammatory bowel disease	United States	Retrospective cohort study	280	White and Black
Tang 2019 [86]	Hepatitis (HBV)	United States	Retrospective cohort study	454	Asian, non-Hispanic White, Hispanic, Black
Thuluvath 2020 [87]	Liver transplant	United States	Retrospective cohort study	154,818	Hispanic, White, Black, Asian, Other
Trooskin 2005 [88]	Hepatitis (HCV)	United States	Cross-sectional study	357	White, Black, Mixed
Volk 2009 [89]	Liver transplant	United States	Retrospective cohort study	50,047	Black, Hispanic, White
Voulgaris 2017 [90]	Hepatitis (HBV)	Greece	Retrospective cohort study	1001	Greek, Immigrants
Warren 2021 [91]	Liver transplant	United States	Retrospective cohort study	30,353	White, Black, Hispanic, Other
Wong 2018 [13]	Hepatitis (HCV)	United States	Retrospective cohort study	29,544	Black, Hispanic
Zheng 2023 [92]	Esophageal disease	United States	Cross-sectional study	9.4 ± 0.3 million	White, Black, Hispanic, Other
Ziv 2022 [93]	Inflammatory bowel disease	United States	Retrospective cohort study	1732	White, Black, Asian, Hispanic, Other

**Table 2:** Racial and ethnic differences in the management of inflammatory bowel disease (IBD).

Author, Year	Comparison Groups	Outcome Measured	Results	*p*-Value
Abadir 2024 [21]	Black vs. White, Asian, LatinX	Delay to initiation of advanced therapy	Longer delay in Black patients: 34 (17–53) vs. White: 19 (10–33), Asian: 17 (13–25), LatinX: 18 (14–49)	<0.001
Abadir 2024 [21]	Distance ≥ 49.5 vs. <49.5 miles	Delay to medication initiation	Longer delay with greater distance: 30 (15–51) vs. 18 (10–33)	<0.001
Abadir 2024 [21]	No insurance approval vs. approved	Delay to medication initiation	Longer delay without approval: 51 (27–84) vs. 18 (11–33)	<0.001
Alchirazi 2022 [22]	Black vs. White	Advanced therapy use (CD)	Lower use in Black patients: adalimumab (OR 0.89), certolizumab (OR 0.81), vedolizumab (OR 0.66), ustekinumab (OR 0.82), tofacitinib (OR 0.58), thiopurines (OR 0.93).	NR
Alchirazi 2022 [22]	Black vs. White	Advanced therapy use (UC)	Lower use in Black patients: infliximab (OR 0.87), adalimumab (OR 0.83), golimumab (OR 0.62), vedolizumab (OR 0.69), ustekinumab (OR 0.73), tofacitinib (OR 0.55).	NR
Alchirazi 2022 [22]	Black vs. White	Healthcare utilization (CD)	Higher odds in Black patients: hospitalization (OR 1.35), obstruction (OR 1.25), intestinal fistula (OR 1.38), perianal abscess (OR 1.56), perianal fistula (OR 1.28).	NR
Alchirazi 2022 [22]	Black vs. White	Healthcare utilization (UC)	Higher odds in Black patients: hospitalization (OR 1.29), toxic megacolon (1.34), C. difficile (1.11). Lower odds of colectomy (0.75).	NR
Barnes 2018 [24]	Black vs. White	Pre-enrollment therapy use	Lower in Black patients: thiopurines in CD (61% vs. 72%), aminosalicylates in UC (80% vs. 89%), methotrexate (0% vs. 7%)	0.003–0.013
Barnes 2018 [24]	Black vs. White	Initiation of anti-TNF (CD)	Higher in Black patients (aOR 1.85, 95% CI 1.09–3.14)	NR
Barnes 2018 [24]	Black vs. White	Therapy use during follow-up	No differences in anti-TNF (UC), combination therapy, thiopurines, methotrexate, or steroids	NR
Barnes 2021 [25]	Black vs. White	Therapy utilization (unadjusted)	Higher use in Black patients: immunomodulators (25% vs. 19%), anti-TNF (25% vs. 20%), combination therapy (5% vs. 3%)	<0.001
Barnes 2021 [25]	Black vs. White	Adjusted therapy use	No difference in anti-TNF; higher combination therapy in CD (aOR 1.50, 95% CI 1.15–1.96)	NR
Barnes 2021 [25]	Black vs. White	Time to anti-TNF initiation	Earlier in Black patients: 52 vs. 104 days	0.027
Barnes 2021 [25]	Black vs. White	Time to immunomodulator initiation	No difference: 33 vs. 64.5 days	0.052
Barnes 2021 [25]	Black vs. White	Time to combination therapy	No difference: 55 vs. 122 days	0.212
Barnes 2021 [25]	Black vs. White	Follow-up duration	Longer follow-up in Black patients: 665 vs. 629 days (median)	<0.001
Benchimol 2016 [26]	Immigrant vs. non-immigrant	Time to diagnosis (CD & UC)	No differences overall (CD HR 1.002; UC HR 1.073)	0.98; 0.27
Benchimol 2016 [26]	Immigrant vs. non-immigrant	ED visits (IBD-specific)	Higher ED use (OR 1.57)	<0.0001
Benchimol 2016 [26]	Immigrant vs. non-immigrant	Outpatient visits	Higher IBD-specific and IBD-related outpatient visits in immigrants	<0.0001; <0.05
Benchimol 2016 [26]	Immigrant vs. non-immigrant	Gastroenterology visits	Higher likelihood of GI visits: IBD-specific (OR 1.37), IBD-related (OR 1.20)	<0.0001
Benchimol 2016 [26]	Latin American/Caribbean vs. others	Time to diagnosis (UC)	Longer time to diagnosis (HR 1.95, 95% CI 1.39–2.73)	0.0001
Benchimol 2016 [26]	GI visit frequency	Surgery risk	Greater GI follow-up associated with lower surgery risk (CD: HR 0.17; UC: HR 0.28)	<0.0001
Branchcomb 2024 [28]	Black, White, Hispanic, Asian, Other	Time to conventional therapy	Black: 24 (4–164); White: 18 (2–132); Hispanic: 18 (2–113); Asian: 15 (5–67); Other: 15 (2–79)	NR
Branchcomb 2024 [28]	Black, White, Hispanic, Asian, Other	Time to advanced therapy	Black: 124 (50–372); White: 139 (43–397); Hispanic: 153 (53–450); Asian: 165 (57–369); Other: 110 (36–380)	NR
Buadu 2024 [29]	Black, Hispanic vs. White	Time to diagnosis	Longer in Black and Hispanic individuals	NR
Chandradevan 2024 [33]	Racial groups	Diagnosis-to-surgery time	No differences between racial groups	NR
Frieder 2023 [42]	Black vs. White	Length of stay (LOS)	Higher odds of extended LOS (OR 1.59, 95% CI 1.45–1.75)	<0.01
Garcia 2024 [44]	Hispanic vs. non-Hispanic	Healthcare utilization	No differences in visits; longer LOS in Hispanic patients	0.001
Garcia 2024 [44]	Hispanic vs. non-Hispanic	Biologic use	No difference (15.8% vs. 19.9%)	0.47
Hussain 2025 [49]	Hispanic vs. NHW	ED visits and admissions	Fewer ED visits (OR 0.7) and admissions (OR 0.6)	<0.0001
Hussain 2025 [49]	Hispanic vs. NHW	Biologic utilization	Higher use (OR 1.3)	<0.0001
Hussain 2025 [49]	Black vs. NHW	ED visits	Higher ED use (OR 1.3)	<0.0001
Hussain 2025 [49]	Black vs. NHW	Steroid utilization	Higher use (OR 1.2)	0.0004
Lin 2013 [54]	Racial/ethnic groups	Immunomodulator & anti-TNF use	No differences observed	NR
McFalls 2025 [60]	Black vs. White	Disease-modifying therapy (6 months UC)	Lower in Black patients (21.3% vs. 26.4%) across all therapy classes	<0.0001
McFalls 2025 [60]	Black vs. White	Pre-diagnosis utilization	Higher ED, inpatient, and endoscopy use	<0.001
McFalls 2025 [60]	Black vs. White	6-month outcomes	Higher ED, hospitalization, mortality	<0.0001
McFalls 2025 [60]	Black vs. White	10-year outcomes	Higher ED, hospitalization, mortality; lower colectomy	<0.0001
McFalls 2025 [60]	Black vs. White	Long-term therapy (10 years)	Persistently lower in Black patients (30% vs. 36%)	<0.0001
McFalls 2025 [60]	Asian vs. White	Healthcare utilization	Higher ED, hospitalizations, endoscopy; fewer colectomies	0.006 to <0.0001
McFalls 2025 [60]	Asian vs. White	Therapy exposure (6 months UC)	Higher overall exposure (34.3% vs. 23.8%), but lower steroids, TNFi, anti-IL-12/23	<0.0001 (varies)
Misra 2019 [63]	White European, Indian, Pakistani	Time to diagnosis and treatment	No differences between groups	NR
Montgomery 2018 [64]	White vs. Black, Asian, Hispanic	Steroid therapy	Higher in White patients (52.7% vs. 50% Black, 49.8% Asian, 47.6% Hispanic)	0.019
Montgomery 2018 [64]	Asian, Hispanic, Black vs. White	Length of stay (LOS)	Longer LOS in minority groups	<0.001; 0.1
Montgomery 2018 [64]	Hispanic vs. others	Emergency surgery	Higher rates in Hispanic patients	<0.01
Montgomery 2018 [64]	Hispanic vs. White	Readmissions	Higher odds (OR 1.4)	0.01
Nguyen 2007 [66]	African American, Hispanic vs. White	Colectomy (UC)	Lower likelihood in African American (OR 0.46, 95% CI 0.35–0.60) and Hispanic patients (OR 0.74, 95% CI 0.59–0.93)	NR
Nguyen 2007 [66]	African American, Hispanic vs. White	Bowel resection (CD)	Lower likelihood in African American (OR 0.68, 95% CI 0.61–0.76) and Hispanic patients (OR 0.70, 95% CI 0.60–0.83)	NR
Nguyen 2007 [66]	African American vs. White	Number of abdominal surgeries (CD)	Fewer surgeries in African American patients: 0.8 vs. 1.6; adjusted difference −0.6 (95% CI −0.9 to −0.2)	<0.001
Nguyen 2007 [66]	African American vs. White	Parenteral nutrition utilization	Lower utilization in African American patients (OR 0.67, 95% CI 0.50–0.89)	NR
Nguyen 2010 [67]	Black vs. White	ED visits	Higher ED visits and proportion with ≥1 visit	0.0001; 0.001
Nguyen 2010 [67]	Black vs. White	Hospitalizations	No differences	0.10; 0.3
Nguyen 2010 [67]	Black vs. White	Ambulatory clinic visits	No difference: 4.7 vs. 4.7 visits/year	0.94
Nguyen 2010 [67]	Black vs. White	Regular GI follow-up (≥1/year)	Lower in Black patients (85% vs. 93%)	0.03
Nguyen 2010 [67]	Black vs. White	Regular IBD specialist follow-up	Lower in Black patients (77% vs. 92%)	0.001
Nguyen 2010 [67]	Black vs. White	Adjusted GI/IBD specialist access	Lower likelihood of regular GI (aOR 0.43) and IBD specialist care (aOR 0.37)	NR
Nguyen 2010 [67]	Black vs. White	Specialist-led care	Lower proportion with IBD specialist as primary provider (46% vs. 72%)	<0.0001
Nguyen 2010 [67]	Black vs. White	Barriers to care	More difficulty with referrals (12% vs. 5%) and cost concerns (18% vs. 7%)	0.02; 0.01
Nguyen 2010 [67]	Black vs. White	Immunomodulator use	Lower in Black patients overall (52% vs. 68%); significant in UC (38% vs. 64%), not CD (59% vs. 70%)	0.006; 0.009; 0.11
Nguyen 2010 [67]	Black vs. White	Prolonged steroid use (>3 months)	Lower in Black patients (72% vs. 87%)	0.003
Nguyen 2010 [67]	Black vs. White	Infliximab use (CD)	Lower in Black patients (41% vs. 60%)	0.01
Odufalu 2024 [69]	Racial groups	ED visits	No differences	0.09
Rao 2024 [71]	Black vs. White	Time to authorization	Longer in African American patients: 45.9 vs. 24.8 days	0.044
Rao 2024 [71]	Racial groups	Time to first infusion (TFI)	No differences across racial groups	0.575
Rao 2024 [71]	Biologic type	Time to first infusion (TFI)	No differences by biologic	0.183–0.812
Segura 2025 [77]	Asian, Hispanic, Black vs. White	Advanced therapy use (pediatric)	Lower in Asian (OR 0.69) and Hispanic (OR 0.74); nominally lower in Black patients	NR
Segura 2025 [77]	Hispanic vs. non-Hispanic	Steroid use & GI access	Lower steroid use (OR 0.88) and fewer GI visits (OR 0.84)	0.098; 0.013
Segura 2025 [77]	Black vs. White (≥65 years)	Advanced therapy use	Lower use in Black patients after adjustment	NR
Segura 2025 [77]	Black vs. White	Healthcare utilization	Higher utilization attenuated after SES adjustment	NR
Segura 2025 [77]	Black vs. White (≥65 years)	ED and steroid use	Higher ED use; lower steroid use	0.001; <0.001
Segura 2025 [77]	Asian vs. White (≥65 years)	Therapy utilization	Lower advanced therapy (OR 0.59), immunomodulators (OR 0.67), steroids (OR 0.62)	NR
Segura 2025 [77]	Asian vs. White	Gastroenterology visits	Lower likelihood of GI visits (OR 0.84)	0.013
Segura 2025 [77]	Asian vs. White (≥65 years)	GI office visits	Lower GI visits in older Asian adults (OR 0.85)	0.139
Shah 2024 [78]	BIPOC/H vs. White	Symptom control & HRQOL	Worse symptom control and HRQOL	0.02 to <0.001
Shah 2024 [78]	BIPOC/H vs. White/non-Hispanic	Access to IBD specialist	Greater difficulty accessing specialist (26% vs. 11%)	0.03
Shah 2024 [78]	BIPOC/H vs. White/non-Hispanic	ED use for IBD	Higher reliance on ED care (42% vs. 22%)	0.004
Shah 2024 [78]	BIPOC/H vs. White/non-Hispanic	Emotional and community support	More difficulty accessing emotional support (42% vs. 18%) and community support (29% vs. 17%)	<0.001; 0.05
Shah 2024 [78]	BIPOC/H vs. White	Access to care	No differences	NR
Syal 2017 [85]	Black vs. White	Readmissions	Higher 30-day and overall readmissions	0.005; 0.018
Syal 2017 [85]	Black vs. White	Length of stay	No difference	0.36
Syal 2017 [85]	Black vs. White	Steroid use (hospitalization)	Higher in White patients	0.001
Ziv 2022 [93]	Racial/ethnic groups	Inpatient therapy utilization	No differences in medications, imaging, or procedures	NR

NR refers to results where the *p*-value was not reported.

**Table 3:** Racial and ethnic differences in the management of disorders of the gut–brain interaction (DGBI).

Author, Year	Comparison Groups	Outcome Measured	Results	*p*-Value
Martinez 2023 [56]	Non-White vs. White	Patient-reported symptom management	Lower likelihood of feeling concerns were fully addressed in non-White patients (OR 0.09)	0.02
Mills 2011 [61]	Non-White vs. White	Pain severity	Higher rates of severe pain (42% vs. 36%)	<0.0001
Mills 2011 [61]	Non-White vs. White	Analgesia use	Lower likelihood of receiving any analgesia (59% vs. 66%) and opiates (39% vs. 51%)	<0.0001
Mills 2011 [61]	Non-White vs. White	Time to analgesia	Longer wait for opiate analgesia (98 vs. 90 minutes)	0.004
Mills 2011 [61]	Non-White vs. White	Admission rates	Lower likelihood of admission (63% vs. 80%)	<0.0001
Silvernale 2021 [81]	Hispanic vs. White	Gastroenterology consultation	Lower GI consult rates (39.0% vs. 69.5%)	<0.01
Silvernale 2021 [81]	Black vs. White	Primary care visits	More IBS-related primary care visits in Black patients	0.0021
Silvernale 2021 [81]	Black vs. White	Gastroenterology consultation	Lower GI consult rates (28.7% vs. 59.3%)	<0.01
Silvernale 2021 [81]	Asian vs. White	Primary care visits	More IBS-related primary care visits in Asian patients	0.039
Silvernale 2021 [81]	Asian vs. White	Gastroenterology consultation	Lower GI consult rates (17.9% vs. 62.7%)	<0.01
Silvernale 2021 [81]	Hispanic vs. White	GI procedures	Higher rates of GI procedures (71.8% vs. 49.4%) and colonoscopy (44.8% vs. 35.8%)	<0.01
Silvernale 2021 [81]	Black vs. White	GI procedures	Higher procedure use (77.7% vs. 50.5%) and colonoscopy (48.6% vs. 34.3%)	<0.01
Silvernale 2021 [81]	Asian vs. White	GI procedures	Higher procedure use (62.8% vs. 45.4%) and colonoscopy (31.6% vs. 23.6%); no difference in EGD (25.0% vs. 21.4%)	<0.01; 0.007; 0.20
Simons-Linares 2018 [82]	Hispanic vs. White	Time to clinical assessment	Higher likelihood of delayed assessment (~30% increase)	0.032

**Table 4:** Racial and ethnic differences in the management of hepatitis.

Author, Year	Comparison Groups	Outcome Measured	Results	*p*-Value
May 2016 [59]	Hispanic vs. Black	Total hospital costs (THC)	Higher costs in Hispanic patients ($42,387 vs. $33,359)	<0.01
May 2016 [59]	Hispanic vs. White	Total hospital costs (THC)	Higher costs in Hispanic patients (1.25-fold increase; 95% CI 1.01–1.49)	NR
May 2016 [59]	White vs. Black	Total hospital costs (THC)	Higher costs in White patients	<0.01
May 2016 [59]	White vs. Black, Hispanic	Length of stay (LOS)	Longer LOS in White patients (6.5 days vs. 5.4 days in Black patients and 5.9 days in Hispanic patients)	<0.01; 0.01
May 2016 [59]	White vs. Black	Number of inpatient procedures	Higher number in White patients (1.3 vs. 1.1)	<0.01
May 2016 [59]	White vs. Hispanic	Number of inpatient procedures	No difference (1.3 vs. 1.3)	0.16
May 2016 [59]	Black vs. White, Hispanic	Inpatient mortality	Lower mortality in Black patients (2.3%) vs. White (4.5%) and Hispanic (3.9%)	<0.01
Buti 2020 [30]	Spanish-born vs. foreign-born	Late presentation to care	Higher late presentation in Spanish-born patients (17% vs. 7%)	<0.0001
Bzowej 2009 [31]	Asian American, Caucasian, African American	Waitlist mortality (1-, 3-, 5-year)	Asian: 3%, 3%, 12%; Caucasian: 10%, 18%, 23%; African American: 6%, 31%, 31%	0.15
Bzowej 2009 [31]	Asian American vs. Caucasian	Waitlist mortality (all-cause)	Lower mortality in Asian Americans after adjustment	0.05
Bzowej 2009 [31]	Asian American vs. Caucasian	Waitlist mortality (liver-related)	No difference after adjustment	0.14
Cooper 2017 [36]	Immigrant vs. Canadian-born	FibroScan utilization	Higher use in immigrants (42% vs. 32%); higher adjusted access across models	<0.001
Cooper 2017 [36]	Immigrant vs. Canadian-born	Biopsy and/or FibroScan	Higher use in immigrants (72% vs. 66%); adjusted OR 1.52 (95% CI 1.13–2.05)	0.008
Cooper 2017 [36]	Immigrant vs. Canadian-born	HCV treatment initiation	No difference (49% vs. 45%)	0.17
Cooper 2017 [36]	Immigrant vs. Canadian-born	Treatment type	No difference between DAA and IFN-based therapy	NR
Elnaiem 2025 [39]	Treated vs. untreated	Specialist linkage (GI/ID clinics)	Untreated patients less likely to be seen in GI (4.7% vs. 24.3%) and ID clinics (5.9% vs. 14%)	<0.001
Elnaiem 2025 [39]	Black vs. White	Treatment initiation	Lower odds in Black patients (aOR 0.68, 95% CI 0.53–0.88)	NR
Elnaiem 2025 [39]	Untreated vs. treated	Healthcare utilization (ED and inpatient encounters)	Higher ED encounters (17.7% vs. 4.8%) and inpatient encounters (12.3% vs. 3.4%) in untreated individuals	<0.001
Giordano 2009 [45]	Immigrant vs. Canadian-born	Liver biopsy	No difference (54% vs. 51%)	Non-significant
Giordano 2009 [45]	Immigrant vs. Canadian-born	HCV treatment initiation	No difference (37% vs. 38%)	Non-significant
Giordano 2009 [45]	Time in Canada	Treatment initiation	No association (209 months in both groups)	0.99
Giordano 2009 [45]	White vs. Black	HCV treatment initiation	Trend toward higher initiation in White patients (38% vs. 29%)	Non-significant
Giordano 2009 [45]	Immigrant vs. Canadian-born	Erythropoietin use	No difference (13% vs. 18%)	Non-significant
Gomes 2020 [46]	Racial/ethnic groups	Linkage to care and retention (HCV)	No differences in linkage or retention rates	NR
Gomes 2020 [46]	African American vs. non-Hispanic White	HCV treatment (post-linkage)	Lower treatment in African American patients (70.4% vs. 74.4%)	<0.05
Gomes 2020 [46]	African American vs. non-Hispanic White	Time from linkage to treatment	Longer delay (280 vs. 165 days)	<0.05
Hu 2013 [48]	Asian/Pacific Islander (foreign-born vs. US-born)	HBV testing rates	Higher testing in foreign-born (48% vs. 31%)	<0.001
Hu 2013 [48]	Race/ethnicity	HBV testing likelihood	African American OR 0.88 (0.77–1.00); Hispanic OR 0.95 (0.86–1.05); Asian/Pacific Islander OR 2.01 (1.80–2.25); American Indian/Alaska Native OR 0.62 (0.31–1.24)	NR
Jiang 2023 [50]	Pre-DAA vs. Post-DAA	HCV treatment initiation	Increased overall initiation (12.6% vs. 7.8%)	<0.001
Jiang 2023 [50]	Black vs. White	Treatment initiation	Lower pre-DAA (aOR 0.64); no significant difference post-DAA (aOR 0.87)	NR
Jiang 2023 [50]	Hispanic vs. White	Treatment initiation	Lower in both pre-DAA (aOR 0.86) and post-DAA (aOR 0.88) periods	NR
Jiang 2023 [50]	Black vs. White	Change over time	Greater increase in treatment initiation in Black patients	0.002
Jiang 2023 [50]	Hispanic vs. White	Change over time	No difference	0.09
Kudaravalli 2024 [52]	Non-Asian vs. Asian	Adequate evaluation	Higher in non-Asian patients (77.8% vs. 73.1%); aOR 1.57 (95% CI 1.25–1.96)	0.01; <0.001
Kudaravalli 2024 [52]	West vs. East	Adequate evaluation	Lower in Western regions (69.0% vs. 77.4%); aOR 0.60 (95% CI 0.55–0.65)	<0.001
Kudaravalli 2024 [52]	Asian (West vs. East)	Adequate evaluation	Lower in Western Asian patients (aOR 0.60, 95% CI 0.55–0.65)	<0.001
Kudaravalli 2024 [52]	Non-Asian (West vs. East)	Adequate evaluation	No difference (aOR 0.95, 95% CI 0.76–1.19)	0.64
Kudaravalli 2024 [52]	West vs. East	NA therapy use	Lower treatment in Western regions (76.3% vs. 86.9%)	<0.001
Kudaravalli 2024 [52]	Asian (West vs. East)	NA therapy use	Lower in Western Asian patients (75.5% vs. 86.9%; aOR 0.54, 95% CI 0.44–0.66)	<0.001
Kudaravalli 2024 [52]	Non-Asian (West vs. East)	NA therapy use	No difference (aOR 0.83, 95% CI 0.46–1.47)	0.51
Kudaravalli 2024 [52]	Asian vs. non-Asian	NA therapy use	No difference (83.3% vs. 84.2%)	0.82
Kudaravalli 2024 [52]	Ethnicity (adjusted)	Antiviral therapy use	No association (aOR 1.54, 95% CI 0.86–2.75)	0.15
Le 2022 [53]	African-born vs. Asian-born immigrants	Linkage to care (HBV)	Lower linkage in African-born immigrants (aOR 0.54, 95% CI 0.28–1.05)	0.069
Le 2022 [53]	Racial/ethnic groups	Linkage to care (HCV)	No differences observed	NR
Miquel 2020 [62]	Immigrant vs. non-immigrant	Liver biopsy	Lower in immigrants (16% vs. 24%)	0.002
Miquel 2020 [62]	Immigrant vs. non-immigrant	Treatment among eligible patients	Lower in immigrants (57.8% vs. 83.2%)	<0.001
Tang 2019 [86]	Asian vs. non-Asian	Linkage to care	Higher linkage in Asian patients (69.1% vs. 47.5%; OR 2.43, 95% CI 1.41–4.18)	<0.01
Tang 2019 [86]	African American vs. non-African American	Linkage to care	Lower linkage in African American patients (35.9% vs. 68.5%; OR 0.33, 95% CI 0.16–0.65)	<0.01
Tang 2019 [86]	African vs. non-African	Linkage to care	No difference (57.1% vs. 68.6%; OR 0.63, 95% CI 0.24–1.63)	0.34
Trooskin 2005 [88]	Black and Hispanic vs. White	HCV testing	Lower in Black (8%) vs. White (11%) and Hispanic (20%); race significant predictor	0.03
Voulgaris 2017 [90]	Greek (non-immigrant) vs. immigrant	Diagnostic monitoring (ALT, CBC, HBV DNA, ultrasound)	Higher monitoring in Greek patients (ALT, CBC, HBV DNA, ultrasound)	0.014–<0.001
Voulgaris 2017 [90]	Greek vs. immigrant	Complete diagnostic classification	Higher in Greek patients (68% vs. 55%)	<0.001
Voulgaris 2017 [90]	Immigrant (2007–2011 vs. 2002–2006)	ALT monitoring	Increased ≥2 ALT measurements over time (29.6% vs. 18.9%)	0.043
Voulgaris 2017 [90]	Greek vs. immigrant	Other diagnostics	No differences in biopsy, liver stiffness, or HBsAg measurement	NR
Voulgaris 2017 [90]	Greek (non-immigrant) vs. immigrant	Loss to follow-up (1 year)	Higher loss to follow-up in immigrants (62.5% vs. 51.5%)	<0.001
Voulgaris 2017 [90]	Greek vs. immigrant	Follow-up beyond 1 year	No difference (80% vs. 77%)	0.342
Voulgaris 2017 [90]	Greek (non-immigrant) vs. immigrant	Treatment rates	Higher in Greek patients (36% vs. 18%)	<0.001
Voulgaris 2017 [90]	Greek vs. immigrant (with indication)	Treatment among indicated patients	Higher in Greek patients (86% vs. 65%)	<0.001
Wong 2018 [13]	Hispanic vs. White	HCV treatment	Lower likelihood in Hispanic patients (OR 0.48, 95% CI 0.39–0.60)	<0.001
Wong 2018 [13]	Asian/Native American vs. White	HCV treatment	Higher likelihood (OR 1.43, 95% CI 1.22–1.67)	<0.001
Wong 2018 [13]	Hispanic vs. White (post-DAA)	HCV treatment	Persistently lower likelihood (OR 0.36, 95% CI 0.26–0.52)	<0.0001

NR refers to results where the *p*-value was not reported.

**Table 5:** Racial and ethnic differences in access to and outcomes of liver transplantation.

Author, Year	Comparison Groups	Outcome Measured	Results	*p*-Value
Ayuk-Arrey 2025 [23]	Black, Hispanic vs. White patients	Listing-to-death ratio (LDR)	Black patients (0.15) had lower LDR than White (0.21) and Hispanic (0.22) patients; Black patients had a lower LDR across all transplant regions.	NR
Chahal 2021 [32]	First Nation vs. Others	Death on the waitlist	Greater rate of death on the waitlist (10.6%) compared to all others referred for a transplant.	0.03
Chahal 2021 [32]	South Asian vs. White vs. First Nations vs. Other patients	Transplant rate	South Asian and White patients had the highest transplant rate (34.5%); First Nations patients had the lowest (30.6%).	0.01
Chahal 2021 [32]	South Asian vs. White vs. First Nations vs. Other patients	Time to transplant	South Asian patients had the shortest time to transplant (197 days) and Asian patients the longest (285 days); no significant differences after competing-risk analysis.	Non-significant
Chatterjee 2023 [34]	Hispanic vs. White patients	Referral phase duration	Hispanic patients spent longer in the referral phase (32.4 vs. 26.0 days).	0.01
Chatterjee 2023 [34]	Hispanic vs. White patients	Evaluation phase duration	Hispanic patients spent longer in the evaluation phase (76.6 vs. 51.6 days), but the difference was not significant.	NS
Cholankeril 2018 [35]	Black vs. Hispanic vs. White patients	Transplant rate	Black patients had the highest transplant rate (62.2%) and Hispanic patients had the lowest transplant rate (44.5%)	<0.001
Cholankeril 2018 [35]	Black vs. White vs. Hispanic	Waitlist removal	Hispanic patients had the highest rate of removal because of clinical deterioration.	<0.001
Cholankeril 2018 [35]	Hispanic vs. Other patients	Waitlist mortality	Hispanic patients had highest waitlist mortality (20.8%) and higher cumulative mortality vs. White patients.	<0.001
Cuomo 2024 [37]	Black vs. non-Black patients	Time to cirrhosis diagnosis	Black patients had a longer time to diagnosis; non-significant after adjustment.	0.03; non-significant after adjustment
Cuomo 2024 [37]	Multiracial vs. Non-multiracial patients	Time to cirrhosis diagnosis	Multiracial patients had a longer time to diagnosis; the association remained significant after adjustment.	0.01; adjusted <0.01
Cuomo 2024 [37]	Hispanic vs. Non-Hispanic patients	Time to cirrhosis diagnosis	Non-Hispanic patients had a shorter time to diagnosis than Hispanic patients.	0.01; non-significant after adjustment
Cuomo 2024 [37]	Hispanic vs. non-Hispanic	Time to death post-cirrhosis	Hispanic ethnicity associated with shorter time to death (HR 0.80; HR 0.82 adjusted).	*p* < 0.01; adjusted 0.02
Cuomo 2024 [37]	White vs. others	Time to death post-cirrhosis	White race showed longer time to death (HR 0.86), but this was non-significant.	Non-significant
Deutsch-Link 2023 [38]	Black vs. Hispanic vs. Asian vs. White patients	Waitlisting (psychosocial exclusion)	Black and Hispanic patients were more likely not to be waitlisted because of psychosocial factors, whereas Asian patients were less likely than White patients.	NR
Goyes 2021 [47]	Hispanic vs. other patients	Waitlist removal (death/deterioration)	Hispanic patients had higher risk of removal (SHR 1.23).	<0.001
Kaplan 2023 [51]	Black vs. White patients	Listing-to-death ratio (LDR)	In alcoholic liver disease, the LDR for Black patients (0.13) was significantly lower than that of White patients (0.26).	<0.001
Kaplan 2023 [51]	Asian vs. White patients	Listing-to-death ratio (LDR)	In alcoholic liver disease, the LDR for Asian patients (0.32) was greater than that of White patients (0.26).	0.004
Kaplan 2023 (ALD) [51]	Black vs. White patients	Transplant-to-listing ratio	Black patients had the highest ratio (0.51), not significantly different from White patients (0.49).	Non-significant
Kaplan 2023 (ALD) [51]	Hispanic vs. White patients	Transplant-to-listing ratio	Hispanic patients had a lower ratio (0.44).	0.02
Kaplan 2023 (non-ALD) [51]	Black vs. White patients	Transplant-to-listing ratio	Black patients had the highest ratio (0.55), not significantly different from White patients (0.53).	Non-significant
Kaplan 2023 (non-ALD) [51]	Hispanic vs. White patients	Transplant-to-listing ratio	Hispanic patients had a lower ratio (0.47).	<0.001
MacConmara 2021 [55]	Minority vs. White patients	Waitlist removal due to death	Minority patients were more likely to be removed from the waitlist because of death (25% vs. 21%) throughout the COVID-19 pandemic.	0.026
MacConmara 2021 [55]	Minority vs. White patients	Waitlist removal due to becoming too sick	No significant difference in removal because patients became too sick for transplant.	Non-significant
MacConmara 2021 [55]	Minority vs. White patients (COVID-19 period)	Waitlist inactivation	Minorities had higher waitlist inactivation during the pandemic; disparities improved over time but overall remained higher.	≤0.001
MacConmara 2021 [55]	Minority vs. White patients (COVID-19 period)	Transplant incidence and listing	Minorities had greater decrease in listing/transplant during pandemic; disparities improved over time but overall remained higher.	≤0.001
Mathur 2010 [57]	Black vs. White patients	Waitlist mortality	Black patients had lower waitlist mortality than White patients (HR 0.63).	<0.0001
Mathur 2010 [57]	Asian vs. White patients	Waitlist mortality	Asian patients had lower waitlist mortality than White patients (HR 0.73).	0.007
Mathur 2010 [57]	Hispanic vs. White patients	Waitlist mortality	No significant difference in waitlist mortality.	Non-significant
Mathur 2014 [58]	Black vs. White patients	Liver wait-listing ratio (acute liver failure)	Black patients had a 25–30% lower liver wait-listing ratio than White patients.	<0.0001
Mathur 2014 [58]	Asian and Native American/Other vs. White patients	Liver wait-listing ratio (acute liver failure)	Asian and Native American/Other patients had higher liver wait-listing ratios in the MELD era than in the pre-MELD era.	0.02
Mathur 2014 [58]	Black vs. White patients	Liver wait-listing ratio (chronic liver failure)	Black patients were waitlisted approximately half as often as White patients.	<0.0001
Mathur 2014 [58]	Hispanic vs. White patients	Liver wait-listing ratio (chronic liver failure)	Hispanic patients had liver wait-listing ratios similar to White patients.	<0.0001
Mathur 2014 [58]	Asian vs. White patients	Liver wait-listing ratio (chronic liver failure)	Asian patients had approximately 50% higher liver wait-listing ratios than White patients.	<0.0001
Mathur 2014 [58]	Native American/Other vs. White patients	Liver wait-listing ratio (chronic liver failure)	Native American/Other patients had the lowest liver wait-listing ratio.	0.056 (pre-MELD); 0.065 (MELD)
Mustian 2019 [65]	Older Black vs. older White patients	Transplant incidence	Older Black patients were less likely to undergo transplantation than older White patients (sdHR 0.92, 95% CI 0.85–1.00).	NR
Mustian 2019 [65]	Older Black vs. younger White patients	Delisting	Older Black patients were more likely to be delisted (sdHR 1.25, 95% CI 1.12–1.40).	0.0001
Mustian 2019 [65]	Older Black vs. younger White patients	Relisting after delisting	Older Black patients were less likely to be relisted (sdHR 0.34, 95% CI 0.18–0.61).	0.0004
Mustian 2019 [65]	Younger Black vs. younger White patients	Waitlist mortality	Younger Black patients had lower waitlist mortality (sdHR 0.88, 95% CI 0.80–0.96).	0.01
Mustian 2019 [65]	Older Black vs. older White patients	Waitlist mortality	Older Black patients had lower waitlist mortality (sdHR 0.85, 95% CI 0.74–0.99).	NR
Mustian 2019 [65]	Older Black vs. older White patients	Mortality after delisting	Not statistically significant.	NR
Mustian 2019 [65]	Black vs. White patients	Time to death after delisting	Time to death was shorter among younger and older Black patients after delisting.	NR
Mustian 2019 [65]	White vs. Black vs. Hispanic patients	Living-donor liver transplantation (LDLT) receipt	LDLT recipients were more likely to be White and less likely to be Black or Hispanic; racial and ethnic minority patients were less likely to receive LDLT.	NR
Nobel 2015 [68]	White vs. Black vs. Hispanic patients	Living Donor Liver Transplantation (LDLT) receipt	LDLT recipients more likely White and less likely Black or Hispanic; racial/ethnic minorities less likely to receive LDLT.	<0.001
Reid 2004 [72]	Black vs. White patients	Transplant receipt	Fewer Black patients received transplant within 4 years than White patients (67% vs. 74.2%), and were less likely to receive transplant even after controlling for multiple confounders.	0.003
Reid 2004 [72]	Black vs. White patients	Waitlist mortality	Black patients were more likely to die or become too sick for transplant (25.6% vs. 17.8%).	<0.001
Reid 2004 [72]	Black vs. White patients	Time to death or becoming too sick for transplant	Black patients had a shorter time to death or becoming too sick for transplant (HR 1.36).	<0.001
Reid 2004 [72]	Black vs. White patients	Time to death or becoming too sick within 4 years	Black patients had higher odds of death or becoming too sick within 4 years of listing (OR 1.52).	0.003
Robinson 2018 [73]	Black vs. White patients	Variceal screening	Black patients were less likely to receive variceal screening (HR 0.34).	<0.01
Robinson 2018 [73]	Black vs. Hispanic and Asian vs. White patients	Variceal screening	All minority groups had lower variceal screening rates within 6 months of diagnosis than White patients.	<0.05
Rocha 2015 [74]	Indo-Asian vs. Other patients	Hospital length of stay	Indo-Asian patients had longer hospital stays.	NR
Rocha 2015 [74]	Indo-Asian vs. Other patients	ICU duration	No difference in duration of intensive care support.	Non-significant
Rocha 2015 [74]	Indo-Asian vs. White patients	Post-transplant survival	No difference at 30 days; worse survival at 1-, 2-, and 5-years overall and in acute liver failure (ALF); not significant for chronic liver failure (CLF).	0.012; 0.003 (ALF); non-significant for CLF
Rosenblatt 2021 [75]	Black vs. White vs. Asian vs. Hispanic patients	Transplant-to-listing ratio	Black patients had the highest ratio (0.53), followed by White (0.49), Asian (0.46), and Hispanic patients (0.46).	<0.01
Rosenblatt 2021 [75]	Black, Asian, Hispanic vs. White patients	Listing-to-death ratio (LDR)	Black patients had the lowest LDR (0.30).	<0.001
Rosenblatt 2021 [75]	Black, Asian, Hispanic vs. White patients	Transplant-to-death ratio	Asian patients had the highest transplant-to-death ratio.	<0.001
Rush 2017 [76]	Hispanic vs. Non-Hispanic patients	Referral to palliative care	Hispanic patients had lower odds of referral to palliative care (OR 0.77).	<0.01
Smith 2017 [83]	Black vs. White patients	Transplant rate	34.9% of White vs. 32.2% of Black patients on waiting list transplanted; no significant difference.	Non-significant
Smith 2017 [83]	Black vs. White patients	Platelet transfusion	Black patients received fewer platelet transfusions.	0.0485
Smith 2017 [83]	Black vs. White patients	Length of stay	Black patients had a shorter hospital stay.	0.0291
Smith 2017 [83]	Black vs. White patients	Rejection rate	Black patients had a lower rejection rate.	0.0279
Smith 2017 [83]	Black vs. White patients	Mortality	No difference in overall mortality.	Non-significant
Smith 2017 [83]	Black vs. White patients	Time to death	White patients had a shorter time to death.	0.0087
Smith 2017 [83]	Black vs. White patients	Follow-up duration	Average follow-up similar between groups (Black patients 4.1 ± 0.3 vs. White patients 3.6 ± 0.2 years).	Non-significant
Solano 2024 [84]	Black vs. White patients	Survival (days alive outside hospital)	Black patients had fewer days alive outside the hospital.	NR
Solano 2024 [84]	Black vs. White patients	Transplant-free survival	No difference in transplant-free survival.	Non-significant
Solano 2024 [84]	Black vs. White patients	TIPS procedure received	Black patients were less likely to receive TIPS (HR 0.40).	NR
Solano 2024 [84]	Different racial/ethnic groups	Paracentesis frequency	No differences in number of paracenteses per person-year across groups.	Non-significant
Thuluvath 2020 [87]	Hispanic vs. White vs. Other patients	Waitlist removal	Hispanic patients were more likely to be removed because of death or clinical deterioration.	<0.01
Thuluvath 2020 [87]	Black vs. White patients	Transplant incidence	Black patients were less likely to receive a transplant after adjusting for covariates.	0.003
Thuluvath 2020 [87]	Asian vs. White patients	Transplant incidence	Asian patients were more likely to receive a transplant after adjusting for covariates.	0.004
Thuluvath 2020 [87]	Hispanic vs. White patients	Transplant incidence	Hispanic patients were the least likely to receive a transplant after adjusting for covariates.	<0.001
Thuluvath 2020 [87]	Asian vs. Hispanic vs. White patients	Mean post-transplant mortality	Asian patients had the longest mean survival (12.1 ± 0.13 years), followed by Hispanic patients (11.2 ± 0.08) and White patients (10.8 ± 0.04).	NR
Thuluvath 2020 [87]	Hispanic vs. Non-Hispanic patients	Survival rate	Compared to Non-Hispanic patients, Hispanic patients had a significantly higher survival rate.	*p* < 0.001
Thuluvath 2020 [87]	Asian vs. Hispanic vs. White patients	Post-transplant mortality	Asian (aHR 0.71) and Hispanic (aHR 0.90) patients had lower mortality risk post-liver transplant compared to White patients, after adjusting for confounders.	<0.0001
Thuluvath 2020 [87]	Black vs. White patients	Post-transplant mortality	Black (aHR 1.27) patients had the highest risk of death compared to White patients, after adjusting for confounders.	<0.0001
Volk 2009 [89]	Black vs. White patients	Transplant and death/removal hazard	Black patients initially higher transplant & death/removal rates, but non-significant after controlling in multivariable analysis.	Non-significant after adjustment
Volk 2009 [89]	Hispanic vs. White patients	Transplant hazard	Hispanic patients were less likely to receive a transplant (HR 0.80); the association disappeared after regional adjustment.	Non-significant after adjustment
Warren 2021 [91]	Black vs. White vs. Hispanic patients	Waitlist representation	Black and Hispanic patients underrepresented on waitlists; White patients overrepresented.	<0.05
Warren 2021 [91]	Black vs. White vs. Hispanic patients	Transplant proportion	In most centers, the percentage of Black patients transplanted exceeded their waitlist proportion, and Black patients were 9.8% of transplants vs. 7.9% of the waitlist.	<0.05

NR refers to results where the *p*-value was not reported.

## Data Availability

The original contributions presented in this study are included in the article/Appendix A. Further inquiries can be directed to the corresponding author.

## Abbreviations

The following abbreviations are used in this manuscript:

APR	Abdominoperineal resection
DAA	Direct-acting antiviral
DALYs	Disability-adjusted life years
DGBI	Disorders of gut–brain interaction
DNA	Deoxyribonucleic acid
GI	Gastrointestinal
HBV	Hepatitis B virus
HCV	Hepatitis C virus
IBD	Inflammatory bowel disease
IBS	Irritable bowel syndrome
LAR	Lower anterior resection
LDLT	Living-donor liver transplantation
LDR	Listing-to-death ratio
LOS	Length of stay
PBC	Primary biliary cholangitis
POEM	Peroral endoscopic myotomy
THC	Total hospital costs
TIPS	Trans-jugular intrahepatic portosystemic shunt
TNF	Tumor necrosis factor

## Appendix A

### Appendix A.2

**Table A1:** Racial and ethnic differences in the management of hepatobiliary disease.

Author, Year	Comparison Groups	Outcome Measured	Results	*p*-Value
Galoosian 2020 [43]	Non-Hispanic White vs. Black, Hispanic, Asian	PBC hospitalizations	Higher proportion among White patients (57.8%→71.2%) vs. Black (4.1–6.3%), Hispanic (8.6–10.9%), Asian (1.7–2.8%)	<0.001
Galoosian 2020 [43]	Black vs. Non-Hispanic White	In-hospital mortality	Higher mortality in Black patients (OR 1.40, 95% CI 1.16–2.03)	<0.05
Galoosian 2020 [43]	Medicaid vs. private insurance	In-hospital mortality	Higher mortality with Medicaid insurance (OR 1.42, 95% CI 1.00–1.99)	<0.05
Porras Fimbres 2023 [70]	Black vs. NHW	Postoperative complications	Higher unplanned reintubation (0.41% vs. 0.30%)	0.0123
Porras Fimbres 2023 [70]	Black vs. NHW	Prolonged hospitalization (>30 days)	Higher rates in Black patients (0.13% vs. 0.05%)	0.0015
Porras Fimbres 2023 [70]	Black vs. NHW	Length of stay (LOS)	Longer LOS (2.24 ± 4.44 vs. 1.71 ± 3.94 days)	<0.0001
Porras Fimbres 2023 [70]	Black vs. NHW	Time to readmission	Longer time from discharge to readmission (11.49 ± 8.04 vs. 10.87 ± 7.91 days)	0.0309
Porras Fimbres 2023 [70]	Black vs. NHW	Postoperative LOS	Longer postoperative stay (1.33 ± 2.47 vs. 1.05 ± 2.22 days)	<0.0001
Porras Fimbres 2023 [70]	Black vs. NHW	Time to surgery (mean days)	Longer time to surgery in Black patients (0.95 ± 5.18 vs. 0.71 ± 5.72 days)	<0.0001
Porras Fimbres 2023 [70]	Black vs. NHW	Delayed surgery (>1 day)	Higher proportion with TTS > 1 day (19.4% vs. 13.4%); higher adjusted odds (OR 1.23, 95% CI 1.17–1.30)	<0.0001
Porras Fimbres 2023 [70]	Black vs. NHW	Operative timing/severity	More emergent surgeries (14.4% vs. 13.7%) and longer operative time (69 vs. 60 min)	0.0232; <0.0001
Porras Fimbres 2023 [70]	Black vs. NHW	Mortality and major morbidity	No differences in mortality, major morbidity, reoperation, or unplanned readmission	NR
Shenoy 2021 [79]	Black, Hispanic vs. White	ED utilization	Higher ED utilization in Black and Hispanic patients	<0.001
Shenoy 2021 [79]	Black, Hispanic vs. White	Time to cholecystectomy	Longer wait times: Black +4 weeks, Hispanic +2 weeks	<0.001
Shenoy 2022 [80]	Black, Hispanic vs. White	Repeat ED use before cholecystectomy	Higher repeat ED use (Black 32.1%, Hispanic 29.6% vs. White 24.5%)	<0.001
Shenoy 2022 [80]	Black vs. White	Repeat ED visits (adjusted)	Higher odds of ≥1 additional ED visit (aOR 1.36, 95% CI 1.07–1.73)	<0.05
Shenoy 2022 [80]	Black vs. White	Wait time thresholds (>60, >90 days)	Higher odds of delays >60 days (aOR 1.38) and >90 days (aOR 1.55); longer total wait time (aIRR 1.28)	<0.05; <0.001
Shenoy 2022 [80]	Hispanic vs. White	Wait time	Higher odds of wait >90 days and longer total wait times	<0.05
Shenoy 2022 [80]	Insurance (self-pay vs. insured)	Wait time to surgery	Higher odds of delays >60 days (aOR 1.82–2.94) and >90 days (aOR 1.76); longer wait times (aIRR 1.53)	<0.001; <0.01
Shenoy 2022 [80]	Bilingual ZIP code vs. others	Wait time	Higher odds of delays >60 days (aOR 1.30), >90 days (aOR 1.37), and longer total wait times (aIRR 1.14)	<0.001

NR refers to results where the *p*-value was not reported.

### Appendix A.3

**Table A2:** Racial and ethnic differences in the management of esophageal disorders.

Author, Year	Comparison Groups	Outcome Measured	Results	*p*-Value
Farrukh 2021 [41]	South Asian vs. White British	Procedural management (achalasia)	Higher likelihood of POEM (z = −3.12) and Heller’s myotomy (z = −2.04); no difference in pneumatic dilation or botulinum toxin use	<0.01; <0.05
Farrukh 2021 [41]	South Asian vs. White British	Treatment patterns	More surgical management (+8%) and less botulinum toxin use (−6%) in South Asian patients; higher likelihood of POEM in less experienced centres	NR
Farrukh 2021 [41]	South Asian, White British (deprived communities)	Long-term management	Lower likelihood of receiving multiple therapies in both groups in deprived settings	NR
Zheng 2023 [92]	Black, Hispanic, Other vs. White	Delay in care due to cost	Lower likelihood of delaying care due to cost (Black OR 0.81; Hispanic OR 0.58; Other OR 0.64)	NR
Zheng 2023 [92]	Black, Hispanic, Other vs. White	Delay in care due to wait times	Higher likelihood of delaying care due to long wait times (Black OR 1.32; Hispanic OR 1.74; Other OR 1.45)	NR

NR refers to results where the *p*-value was not reported.

### Appendix A.4

**Table A3:** Racial and ethnic differences in the management of acute gastrointestinal disease.

Author, Year	Comparison Groups	Outcome Measured	Results	*p*-Value
Bickell 2008 [27]	White vs. Non-White patients	Time to diagnosis	Longer time to diagnosis and length of stay in the emergency department for non-White patients without insurance	<0.001
Bickell 2008 [27]	White vs. Minority patients	Time to operating room from emergency department	Minority patients were as likely as White patients to leave the emergency department for the operating room in good time, if they had insurance.	NR
Bickell 2008 [27]	White vs. Minority patients	Time to diagnosis	Minority patients (70%) were more likely than White patients (30%) to have a time to diagnosis greater than or equal to 3 hours.	<0.001

NR refers to results where the *p*-value was not reported.
